# Smoking Cessation and Socioeconomic Status: An Update of Existing Evidence from a National Evaluation of English Stop Smoking Services

**DOI:** 10.1155/2015/274056

**Published:** 2015-07-26

**Authors:** Rosemary Hiscock, Fiona Dobbie, Linda Bauld

**Affiliations:** ^1^Department for Health, University of Bath, Bath BA2 7AY, UK; ^2^UK Centre for Tobacco and Alcohol Studies, UK; ^3^Institute for Social Marketing, School of Health Sciences, University of Stirling, Stirling FK9 4LA, UK

## Abstract

Smokers from lower socioeconomic groups are less likely to be successful in stopping smoking than more affluent smokers, even after accessing cessation programmes. Data were analysed from 3057 clients of nine services. Routine monitoring data were expanded with CO validated smoking status at 52-week follow-up. Backwards logistic regression modelling was used to consider which factors were most important in explaining the relationship between SES and quitting. The odds ratio of stopping smoking among more affluent clients, compared with more disadvantaged clients, after taking into account design variables only, was 1.85 (95% CI 1.44 to 2.37) which declined to 1.44 (1.11 to 1.87) when all controls were included. The factors that explained more than 10% of the decline in the odds ratio were age, proportion of friends and family who smoked, nicotine dependence, and taking varenicline. A range of factors contribute to lower cessation rates for disadvantaged smokers. Some of these can be modified by improved smoking cessation service provision, but others require contributions from wider efforts to improve material, human, and social capital.

## 1. Introduction

In this paper, we explore why smoking cessation rates among lower socioeconomic status (SES) smokers are poorer than those of higher SES smokers. The study focuses on adults who accessed stop smoking services in England. To contextualise our work, we first introduce SES and the relationship between SES and health. Using data collected from services, we then explore the relationship between SES and smoking, smoking cessation, and behavioural support.

SES can be described as the position of a person in the structure of society due to social or economic factors [1]. There is no single measure of SES [1] but SES embodies an array of resources: material capital such as money and goods, human capital such as skills, knowledge, prestige or power, and social capital—beneficial social connections [2–4]. Building upon Coleman's Social Theory [4], Oakes and Rossi [3] suggest that SES should be assessed through measures of material capital including measures of income and housing status [3], measures of human capital including measures of education and occupation [3] and measures of beneficial social connections such as measures of marital status and two parents rather than a single parent [1].

These material, human and social resources can be deployed in order to promote health [2, 3]. The theory of fundamental causes [2] posits that SES can be a cause of poor health because it can be persistent over time and it influences multiple disease outcomes, through disease risk factors and mechanisms. These include demographic differences, psychological factors, access to medical care, social environment, exposure to carcinogens and pathogens, CNS and endocrine response, and health behaviours [5]. Health behaviours have been found to be an important mechanism: together, smoking, physical activity, and consuming alcohol have been found to explain 68% [6] of the mortality difference between low SES groups. Tobacco use alone has been found to be responsible for 50 to 65% of the difference in mortality rates based on socioeconomic status [7, 8]. This is because smoking rates are higher among those with lower SES in the majority of developed countries [9] and also in many low and middle income countries [10]. Despite an overall decline in smoking rates in the developed world, declines have been slower or nonexistent amongst disadvantaged groups, thus increasing inequalities [11–18].

Smoking prevalence may decline through lower uptake of smoking and through smoking cessation. However, disadvantaged smokers have higher rates of uptake and lower rates of successfully stopping [19–25] and findings from studies examining SES and intention to quit are inconsistent [23, 26]. It appears that, in England at least, recent declines in prevalence may be more to do with reduced uptake rather than increased quitting with quit rates possibly even declining among more disadvantaged groups [17]. Therefore, in order to improve the chances of current smokers successfully stopping, we need to increase our understanding of why low SES smokers find stopping smoking more difficult and use this to inform policies and interventions. A recent review [10] concludes that some disadvantaged smokers have more stressful lives due to material hardship (or in other terms less material capital): psychological differences and greater dependence on tobacco coupled with less motivation to quit (even when they have embarked on a quit attempt) and a higher rate of smoking among family and friends (or less social capital related to smoking).

SES differences in quit rates have also been observed in studies of smoking cessation interventions [27–29]. Reviews of the evidence suggest that the most promising tobacco control interventions to reduce smoking rates among disadvantaged smokers are fiscal measures, in particular tobacco taxation. However, there is also evidence to suggest that individual level smoking cessation programmes providing a combination of both pharmacotherapy and structured behavioural support have some success among disadvantaged smokers [30–32]. Smoking cessation quit rates are poorer for disadvantaged smokers but this can be mitigated by focusing resources on these communities [30]. In countries where smoking cessation services are widely available and have successfully targeted disadvantaged groups (such as in the UK) [33], inequalities have not yet declined although they may have stabilised [34, 35]. Ideally, however, quit rates would be similar in all social groups so it would be helpful to find out why there are differences between socioeconomic groups taking part in such programmes.

In a previous paper [36] we used data from two longer term evaluations of Stop Smoking Services in the UK to examine the relationship between SES and quitting. The main factor associated with an unsuccessful quit attempt amongst disadvantaged clients, in addition to dependence and social networks, was not adhering to treatment (a stop smoking programme including access to medication and behavioural support). However, it is possible that inclusion of adherence in the model in our study was merely a reflection of quitting given that clients who relapse would tend to stop taking medication and attending sessions; this would imply that adherence differences may be masking underlying reasons for the difference in quit rates between disadvantaged and more affluent smokers. Thus, a new analysis is needed using more recent data.

The purpose of this paper is threefold. First, to explore whether material, human, and social capital-related indicators of SES are associated with smoking cessation amongst clients of smoking cessation services in England, second, to explore which material, human, and social capital related predictors of cessation are associated with SES, and third, to explore whether predictors of cessation explain why there is a relationship between SES and successfully stopping smoking.

## 2. Methods

### 2.1. Data

This paper reports results from a prospective cohort study involving longer term follow-up of smokers accessing Stop Smoking Services in England (ELONS) [37]. All clients were offered behavioural support delivered by trained advisors based on national guidelines and pharmacotherapy (NRT and varenicline) during their quit attempt. About 5% of clients who attended nine services (Bristol, County Durham and Darlington, Hull and East Riding, Leicestershire County and Rutland, North and North East Lincolnshire, Northamptonshire, Oldham, Rotherham and South East Essex) between March 2012 and March 2013 were enrolled. Data from the 3057 clients who were enrolled to the ELONS study were included in the analysis.

Stop smoking service practitioners were asked to recruit clients to the study. If clients consented to be part of the study, monitoring data collection was enhanced compared with normal practice. Clients were asked to set a quit date and if they reported to their practitioner that they were quit at four weeks, they were followed up at 52 weeks by a social research company (TNS-BMRB) through a telephone interview. If they reported they did not quit they were assumed to be relapsed at 52 weeks. Clients lost to follow-up were also coded as smoking in an intention to treat approach.

If clients indicated that they quit they were asked to complete a carbon monoxide (CO) breath test during a home visit and whether they had smoked since their quit date. The outcome variable was CO validated continuous abstinence but clients were allowed to have smoked up to five cigarettes. Data collection complied with the Russell standard commonly used in smoking cessation studies [38–41].

Weighting was undertaken to correct for nonresponse. In order to create the weights, the research team acquired anonymised routine monitoring data on all quit attempts (with quit dates) that took place at the nine study sites from March 2012 to March 2013 (the months where any ELONS client set a quit date). Applying weights enabled quit rates to be generalizable to all clients who were supported by the nine services that took part in ELONS. The weights were trimmed rim weights which were provided by TNS-BMRB and took into account behavioural support type, age, gender, and SES (measured by NS-SEC [42]). Note that the “other/unclear” group of behavioural support in ELONS was too small for weighting so quit attempts in this group were redistributed to either the nearest group or the group of which they were most likely to be a member (three quit attempts to GP practice service and the remainder to one to one specialist). We intended to also weight for location (study site) but there were large differences in proportions recruited by location which led to instability in the weighting and so the decision was made to exclude this. As an alternative, quit rates were calculated taking into account clustering by location. The software used to create these weighted quit rates was Stata [43]. Note that wide confidence intervals resulted from the weighting procedure as found elsewhere when the variance between groups is large [44, 45].

### 2.2. Measures

Two measures of SES were already included in routine monitoring data collection: eligibility for free prescriptions (a measure of income) and economic status (through NS-SEC [42] which codes type of occupation for respondents with a job and reasons for not working for others). Through the enhanced monitoring data, three more SES indicators were collected: housing tenure, highest educational qualification, and household type. These were added because they are common measures of SES and they correspond to material capital, human capital, and social capital, respectively. To derive a composite measure of SES, the five indicators of SES were divided into two categories, one of which included clients who were disadvantaged according to that indicator. The disadvantaged categories were social or private renter, eligible for free prescriptions, routine or manual occupation or unemployed or permanently sick, basic (GCSE) or no educational qualifications, and single parent household. The number of these disadvantaged characteristics possessed by each client was counted. Clients possessed between zero and five of these characteristics. This count of indicators was dichotomised into more affluent (zero to one indicators) and more disadvantaged (two to five indicators). The low cut off point was chosen for two reasons. First, some population groups were only affected by some indicators; for example, all clients aged sixty or more were eligible for free prescriptions and men are less likely to be single parents. Second, this cut point provided adequate sample sizes for analysis.

Demographic variables included age, gender, and ethnicity. Age at first contact was included in the analysis. Ethnicity was categorised as white British, other white, Asian (including mixed white and Asian) and other. To assess wellbeing, following standard practice, the WHO-5 Wellbeing scale [46] items were converted to a percentage. Thus, a score of 0 indicates the lowest wellbeing and a score of 100 represents the highest wellbeing.

Stop Smoking Services' behavioural support provision can either be provided through “specialist” or “level 2” services by trained practitioners. Specialist practitioners are directly employed by the stop smoking services and only provide stop smoking support. Level 2 services involve staff employed by other organisations (chiefly GP practices and pharmacies) who provide stop smoking support alongside their other duties. The behavioural support types analysed were specialist groups, specialist drop ins, specialist one to one, level 2 GP practice/pharmacy service (note this is chiefly one to one), and other/unknown.

Seasonality effects were included because our previous work [47] suggested that the success of quit attempts varied throughout the year with quit attempts in the new year being particularly successful. Such effects were analysed in this analysis through the time of year that a quit attempt started. Quit attempts which started during the main summer holiday period, the postsummer holiday “back to school” period and the new year were differentiated from those starting at other times of year. Medication was operationalised by whether or not clients had taken varenicline at week one. NRT was not differentiated because of multicollinearity with behavioural support (due to site choices of types of medication and behavioural support provision provided). Varenicline at week one was measured because abstinence from smoking was strongly associated with higher numbers of records of smoking medication and clients who had more records had more opportunity to change medication.

Initial analysis of dependence showed that high daily consumption and smoking within five minutes of waking were associated with low quit rates. However, there was not a linear relationship between either of these variables and quitting. Thus, the Heaviness of Smoking Index (HSI) was only of borderline significance in preliminary analysis [37] and concerns arose that the true importance of being dependent might be missed if the HSI was used to represent dependence so instead a dichotomous variable was used: clients who smoked >30 cigarettes per day or who smoked within five minutes of waking were coded as dependent and contrasted with all other clients.

Clients who stated that their spouse or partner was supporting them during their quit attempt were also differentiated from other clients as were clients who indicated that half, a few, or none of their friends and family smoked.

### 2.3. Analysis

#### 2.3.1. Which Markers of SES Predicted Smoking Cessation?

To provide an understanding of the components of our composite measure of SES, weighted quit rates (and 95% confidence intervals) were calculated for each of the five markers of SES: eligibility for free prescriptions, NS-SEC, housing tenure, educational qualifications, and household type.

#### 2.3.2. Which Predictors of Cessation Were Associated with SES?

Elsewhere [37], we have used multivariate logistic regression to model significant predictors of CO validated quitting 52 weeks after clients set a quit date. Here, we have calculated weighted disadvantage rates (and 95% confidence intervals) for each categorical characteristic that predicted quitting and weighted means of more affluent and more disadvantaged clients (and 95% confidence intervals) for the characteristics operationalised through continuous variables. We also present weighted quit rates and weighted means of quitters and nonquitters (and 95% confidence intervals).

#### 2.3.3. Did Any Predictors of Cessation Explain Any of the Relationships between SES and Abstinence from Smoking?

SPSS version 22 was used for regression analysis. Confounders for the relationship between SES and cessation were identified by the following procedure. First, SES was entered alone into a logistic regression model predicting quitting and the odds ratio of more affluent clients (compared to more disadvantaged clients) was noted. Second, design variables (behavioural support type) were added to the model. These reflected differential recruitment and the model would not provide generalizable results without their inclusion. Again the odds ratio of more affluent clients was noted. Third, all other variables that had previously been identified as significant predictors of cessation [37] were entered and the odds ratio of more affluent clients was noted. Then, each significant predictor in the full model was removed in turn and the odds ratio of more affluent clients was noted.

The difference between the odds ratio of more affluent clients in the design variable model and the final model was calculated. The threshold for a variable being a confounder between SES and quitting was set as reducing the difference by more than 10%.

## 3. Results

### 3.1. Which Markers of SES Predict Smoking Cessation?

Quit rates by each marker of SES are presented in Table 1 in order to explore the components of our composite measure of SES. Confidence intervals overlapped for all economic status groups, suggesting differences were not significant; we could not be sure the difference found would be similar in the population, although clients with managerial/professional and intermediate occupations did have higher quit rates. Clients with routine and manual occupations or who were unemployed or permanently sick had similar (lower) quit rates.

Confidence intervals overlapped for all education categories (again suggesting differences were not significant) but quit rates were in the expected direction: clients with basic or no qualifications had the lowest quit rates and clients with A levels or tertiary education had the highest. Clients eligible for free prescriptions had the lowest quit rates. The highest quit rates were for those outside the relevant age group, possibly because older clients, who may also have reached the age where they become eligible for free prescriptions in England, irrespective of income, are more likely to stop smoking. Confidence intervals did not overlap between clients living in rented accommodation and clients who lived in owner occupied housing suggesting that the latter clients were significantly more likely to quit. Confidence intervals overlapped between all household types suggesting no significant differences. Clients who did not answer this question or had a nonspecified household type were least likely to stop smoking followed by single parents. Note that only ten percent of the sample could be categorised as single parents.

Thus, the only marker of SES where confidence intervals between the highest SES group and the lowest SES group did not overlap was housing tenure. The disadvantaged category with the lowest cessation rate was single parents (weighted quit rate 5.1% (95% CI 2.3 to 10.9)) and the affluent category with the highest quit rate was owner occupiers (weighted quit rate 10.8% (95% CI 9.3 to 12.5)).

The cessation rates of the more affluent smokers and disadvantage smokers (from the derived variable where indicators of disadvantage were counted) are presented in Table 2. Confidence intervals of the cessation rates for affluent and disadvantaged smokers did not overlap (more affluent 10.3% (95% CI 8.4 to 12.7) and more disadvantaged 6.2% (95% CI 5.0 to 7.7)) suggesting a significant difference between SES groups.

### 3.2. Which Predictors of Cessation Are Associated with SES?

Before analysis to find intermediate variables on the pathway between SES and smoking cessation, bivariate relationships between each candidate with firstly quitting and secondly SES should be considered. The quit rates and rates of disadvantage for each characteristic are presented in Table 2.

Confidence intervals overlapped, suggesting no significant difference, for the following variables: behavioural support type and location (there were more disadvantaged clients who attended specialist drop-ins and fewer disadvantaged clients among those who attended groups), seasonality (there were fewer disadvantaged clients among those who started their quit attempt in the new year), gender, wellbeing (more affluent clients had higher levels of wellbeing than more disadvantaged clients), medication (fewer disadvantaged clients took varenicline at the start of their quit attempt), and a spouse or partner who supported the quit attempt (fewer disadvantaged clients had a supportive spouse or partner).

Confidence intervals did not overlap, implying significant differences, for the following variables: age (disadvantaged clients tended to be younger), tobacco dependence (disadvantaged clients were more likely to be dependent), and proportion of family and friends (social network) who smoked (disadvantaged clients were less likely to say that half or fewer of their family and friends smoked).

### 3.3. Did Any Predictors of Cessation Explain Any of the Relationships between SES and Abstinence from Smoking?

The odds ratio of stopping smoking for more affluent clients with the design variables only entered besides SES was 1.85 (1.44 to 2.38) (Table 3). This attenuated to 1.44 (1.11 to 1.87) when all other significant predictors of quitting were added. Note that SES is still a significant predictor of cessation. The variables that crossed the threshold suggesting they were important in explaining the relationship between SES and quitting were age (OR of more affluent 1.52 (1.18 to 1.97)), dependence (OR of more affluent 1.50 (1.15 to 1.94)), social network smoking (OR of more affluent 1.50 (1.15 to 1.95)), and varenicline use (OR of more affluent 1.49 (1.15 to 1.93)).

## 4. Discussion

Socioeconomic disadvantage has an important role to play in the differences in success rates in stopping smoking between more and less affluent smokers. This study illustrates that these differences remain even when smokers have access to effective treatment services that are free at the point of use. We discuss here the main findings of the study, as well as some of the limitations of the research.

### 4.1. Main Findings of the Study

In the introduction, we introduced three types of capital pertinent to SES: material capital, human capital, and social capital. We included measures of SES that reflect these. The first area of investigation was the association between these various indicators of SES and cessation. The difference between housing tenures was the most marked of the SES indicators because confidence intervals did not overlap suggesting a significant difference. The two main tenure categories (renting and owner occupation) both contained over 1000 clients and the other/unknown category was one of the smallest. This may have helped strengthen the association. In addition there was a larger gap between the two main tenure categories than the other SES indicators (over four percent gap for tenure compared with less than a two percent gap for education) suggesting the difference was not just due to methodological reasons. Particularly marked differences between housing tenures when compared to other SES indicators has been found elsewhere perhaps because it may reflect cumulative prosperity (wealth over a long time period) [48, 49].

Housing tenure differences appearing more marked than educational differences may suggest that it is material factors that are more important in hindering smoking cessation rather than human capital, cognitive or acquired skill differences between more affluent and more disadvantaged groups. We did not look at cognitive differences in the study but we did look at wellbeing differences. Although wellbeing differences were found in the expected direction they were not as marked as other factors. Our findings may also suggest that material differences were more marked than the social differences (between lone parent and other households, for example). However, the small number of single parents in the study may have reduced the valence of this measure.

In the second part of the study, we considered whether other measured factors might affect the relationship between SES and smoking cessation.

In terms of material capital, even though our method of deriving the composite SES variable was intended to reduce the age bias, age was still the strongest confounder of the relationship between SES and stopping smoking. Internationally, evidence suggests that older people are, in general, wealthier [50] and that younger people were disproportionally affected by the recent global recession [51]. As smokers age the health effects of smoking become more apparent [52] but it might also be the case that younger smokers fail to stop smoking due, at least in part, to the considerable stresses of material hardship.

Human capital includes motivation. In this dataset motivation did not predict cessation in the long term, possibly because more disadvantaged smokers in the study reported higher levels of determination to quit. We found that 64% (95% CI 60 to 68) of more disadvantaged smokers were very or extremely determined to quit compared with 57% (95% CI 51 to 62) of more affluent smokers.

Other human capital-related concepts were important for cessation such as lower dependence on tobacco and taking medication intended to help with stopping smoking. Our results also suggest that more disadvantaged smokers were less likely to quit because they were less likely to be offered or take varenicline (an effective pharmacotherapy) as part of their treatment programme. This may reflect that some of the measures of disadvantage (particularly an economic status of “permanently sick” and qualifying for free prescriptions) are indicators of health disadvantage as well as socioeconomic disadvantage. Smokers with some health conditions have contraindications for varenicline. Conversely, an Australian study using education, income and neighbourhood deprivation as SES indicators found that low socioeconomic status smokers were more likely to take prescription medicine [53]. Another explanation is that practitioners within some services in the study may have been less likely to recommend or offer varenicline to particular groups of clients, although this is not something we were able to explore within the data available to us.

In terms of social capital, the results of the study are similar to previous work that has suggested that having more smokers in social networks (family and friends) may serve as a barrier to smoking cessation [10].

Our analysis of measures of SES suggested factors that reflect material disadvantage may be most important. However, our analysis of factors that confound the association between SES and smoking suggested that human and social issues are also relevant. Although we have split our measures of SES and confounding variables into those that are material, human, and social capital-related, in reality these factors are interlinked. For example, tobacco dependence tends to be higher among smokers with more friends and family who also smoke, perhaps due to higher levels of consumption through time [54].

### 4.2. Limitations

This study faced a number of limitations. We were only able to recruit a small proportion of eligible service clients in each of the nine areas, primarily because of the consent process required for the study which was at odds with staff being able to introduce the research to all the smokers they saw. We therefore attempted to address this recruitment issue by applying weights to the data. Additionally, although the data came from nine contrasting areas of the England, these areas are not necessarily representative of cessation service clients although we deliberately recruited from areas with varying success rates [37].

Less than 10% of quit attempts in the UK involve the use of cessation services [55] and only 5% of clients attending nine services were recruited to the study. Thus the evidence in this paper only applies to a small proportion of smokers. Nevertheless, these services are one of the most cost-effective of all healthcare interventions [56] and evidence of the kind outlined in this paper can contribute to expanding the reach and effectiveness of these programmes.

Asking stop smoking service practitioners rather than researchers to recruit may have resulted in a lower response rate due to competing priorities. However, employing the required number of researchers to cover nine areas would have been expensive and was beyond the scope of the study. In addition, we would ideally have used a more formal mediation analysis but given the need for weighting, a dichotomous outcome and multicategory design variables no such methodology was found to be suitable. Furthermore our comparisons of quit rates and rates of disadvantage are somewhat exploratory because we did not undertake formal tests of differences for similar reasons.

The factors included in the modelling were unable to fully explain the relationship between SES and smoking. In future, studies should compare and contrast material, cognitive, and psychological consequences of deprivation for smoking cessation in order to understand how lower SES smokers can be helped to maintain abstinence from smoking in the long term.

## 5. Conclusion

Findings from this evaluation of longer term outcomes for smokers enrolled in a national cessation service suggest that these types of services face a number of challenges in supporting more disadvantaged smokers to quit. Most of the barriers identified relate to the individual circumstances of these smokers. Services need to be able to identify these factors and, if appropriate, tailor behavioural support to help address them in some measure. In addition, higher levels of tobacco dependence amongst these smokers should be recognised and treated with appropriate provision of and advice around pharmacotherapy including, importantly, use of varenicline where available. There is also an ongoing need to link cessation programmes with wider tobacco control measures that support disadvantaged populations to change behaviour. Some of the more effective policies should focus on how material hardship can be alleviated alongside promoting smoking cessation. Our results also highlight the need for further research, in particular to explore why specific markers of socioeconomic status, such as housing tenure, serve as a predictor of abstinence from smoking after accessing a treatment programme.

## Figures and Tables

**Table 1:** Distribution and CO validated quit rates among SES indicators.

	*N*	%	% CO validated quit at 52 weeks (weighted)
NSSEC–economic status			
Routine and manual occupations^∗^	939	30.7	6.5 (4.7 to 8.9)
Managerial/professional and intermediate occupations	716	23.4	9.3 (6.0 to 14.0)
Sick/disabled and never worked/long term unemployed^∗^	660	21.6	6.8 (4.7 to 9.9)
Other (e.g., retired)/unknown	742	24.3	8.5 (6.6 to 10.8)
Highest educational qualification			
Basic (GCSE) or none^∗^	1452	47.5	7.1 (5.5 to 9.3)
Other (e.g., vocational)/unknown	1000	32.7	8.2 (5.8 to 11.6)
A level or degree	605	19.8	8.6 (6.0 to 12.1)
Eligibility for free prescriptions			
Free^∗^	1433	46.9	6.3 (5.1 to 7.9)
Pays	1080	35.3	8.4 (6.1 to 11.4)
Outside relevant age range (19–59) or unknown	544	17.8	9.7 (7.6 to 12.5)
Housing tenure			
Social/private renting^∗^	1487	48.6	6.1 (4.9 to 7.4)
Other/unknown	316	10.3	4.1 (1.3 to 12.6)
Owner occupier	1254	41.0	10.8 (9.3 to 12.5)
Household type			
Single parent^∗^	309	10.1	5.1 (2.3 to 10.9)
Married/cohabiting and children	664	21.7	9.4 (5.8 to 14.8)
No children in household	1832	59.9	7.9 (7.0 to 8.9)
Other/unknown	252	8.2	4.7 (1.6 to 13.3)
Total	3057	100.0	7.7 (6.6 to 9.0)

^∗^These categories were included in the count of markers of disadvantage.

**Table 2:** ELONS 52-week weighted CO validated quit rates (percents and weighted 95% CI), weighted means of age and wellbeing (and weighted 95% CI) by key variables^∗^.

	*N*	%	% CO validated quit at 52 weeks	% disadvantaged
			(95% CI)	(95% CI)
			(weighted)	(weighted)
SES				
0-1 indicators of disadvantage	1123	36.7	10.3 (8.4 to 12.7)	0
2–5 indicators of disadvantage	1934	63.3	6.2 (5.0 to 7.7)	100
Behavioural support				
Group specialist	652	21.3	12.1 (10.5 to 13.8)	57.5 (46.8 to 67.6)
Drop in specialist	887	29.0	7.6 (5.1 to 11.0)	70.3 (63.7 to 76.2)
One to one specialist	1131	37.0	10.2 (7.6 to 13.7)	64.1 (55.0 to 72.2)
GP practice/pharmacy service	366	12.0	5.1 (2.8 to 9.3)	60.1 (53.7 to 66.2)
Other or unknown	21	.7	Not available	Not available
Time of year of quit attempt				
Other months	767	25.1	7.0 (5.2 to 9.4)	61.7 (55.3 to 67.6)
Summer: July, August	970	31.7	6.3 (4.4 to 8.9)	64.3 (56.4 to 71.5)
Back to school: September, October	1128	36.9	8.7 (6.4 to 11.7)	65.6 (61.1 to 69.8)
New Year: January, February	192	6.3	13.1 (5.1 to 29.6)	51.5 (29.5 to 73.0)
Age (in years)			*Not quit 43.3 (42.5 to 44.1) *	*2–5 disad.* 41.2 (39.7 to 42.7)
(weighted mean)			*Quit 46.8 (44.4 to 49.2) *	*0-1 disad.* 47.7 (45.5 to 49.9)
Gender				
Female	1710	55.9	7.2 (6.0 to 8.5)	64.0 (59.6 to 68.2)
Male	1347	44.1	8.4 (6.8 to 10.2)	62.4 (56.1 to 68.3)
WHO_5 Wellbeing			*Not quit 52.7 (51.4 to 53.9)*	*2–5 disad.* 51.3 (47.5 to 55.2)
(weighted mean)			*Quit 59.3* *(56.5 to 62.1) *	*0-1 disad.* 56.4 (52.5 to 60.3)
Medication in week 1				
Varenicline not recorded	1661	54.3	6.2 (4.9 to 7.7)	66.3 (60.7 to 71.5)
Took varenicline	1396	45.7	10.0 (7.2 to 13.8)	58.8 (50.8 to 66.4)
Dependence				
Other	1681	55.0	9.8 (7.7 to 12.4)	57.9 (53.2 to 62.4)
Highly dependent	1376	45.0	4.9 (2.9 to 8.2)	70.5 (65.9 to 74.7)
Support from spouse partner				
Other	1507	49.3	6.2 (4.5 to 8.5)	67.1 (60.7 to 72.9)
Support from spouse/partner	1550	50.7	9.2 (7.4 to 11.3)	59.6 (54.3 to 64.6)
Friends and family				
Other	771	25.2	3.4 (2.6 to 4.4)	75.2 (66.5 to 82.3)
Half or fewer smoke	2286	74.8	9.1 (7.5 to 10.9)	59.5 (55.3 to 63.6)
Base	**3057**	**100**	**7.7 (6.6 to 9.0)**	**63.3 (58.7 to 67.6)**

^∗^Significant differences in SES but not quitting by location (not shown).

**Table 3:** 52-week adjusted odds ratios (and 95% CI) by key variables and OR (95% CI) of disadvantaged SES is models varying the entry of other variables.

	Adjusted odds ratios in full model	Odds ratio (95% CI) of CO validated quitting for low SES clients (2 to 5 indicators of disadvantage) compared to more affluent clients (0 to 1 indicators of disadvantage)
SES only entered		1.93 (1.51 to 2.47)
Design variable^∗^ model		1.85 (1.44 to 2.38)
SES		
0-1 indicators of low SES	1.4 (1.1 to 1.9)	1.44 (1.11 to 1.87)
2–5 indicators of low SES	1	
Age (in years)^∗^	1.011 (1.002 to 1.020)	**1.52 (1.18 to 1.97)**
Gender		1.43 (1.10 to 1.86)
Female	1	
Male	1.2 (0.9 to 1.5)	
Seasonality		1.43 (1.10 to 1.86)
Other months	1.2 (0.8 to 1.7)	
Summer: July, August	1	
Back to school: September, October	1.2 (0.9 to 1.6)	
New Year: January, February	1.7 (1.0 to 2.9)	
Wellbeing	1.007 (1.0003 to 1.013)	1.47 (1.13 to 1.91)
Dependence		**1.50 (1.15 to 1.94)**
Other	1.5 (1.1 to 1.9)	
Highly dependent	1	
Support from spouse partner		1.47 (1.14 to 1.91)
Other	1.0	
Support from spouse/partner	1.4 (1.0 to 1.8)	
Social network		**1.50 (1.15 to 1.95)**
Other	1.0	
Half or fewer smoke	2.0 (1.4 to 2.9)	
Medication		**1.49 (1.15 to 1.93)**
Varenicline not recorded	1	
Took varenicline	1.7 (1.3 to 2.3)	

^∗^Design variable model includes behavioural support type and location due to differential recruitment.

Bold area indicates variable passed the threshold (calculated as 1.48) of being relevant in the relationship between SES and CO validated quitting.
